# Automatic Recognition and Quantification of Multiple Defects in Highway Tunnels Using Vehicle-Mounted Multisensor Inspection

**DOI:** 10.3390/s26144378

**Published:** 2026-07-10

**Authors:** Yipeng Liu, Jianyu Hong, Xuezeng Liu

**Affiliations:** 1 College of Mechanical and Electrical Engineering, Lanzhou University of Technology, Lanzhou 730050, China; 2 College of Civil Engineering, Tongji University, Shanghai 200092, China

**Keywords:** highway tunnel, defect recognition, multisensor inspection, defect quantification

## Abstract

With advances in computer vision and modern surveying technologies, intelligent inspection systems and automatic recognition methods are increasingly used in highway tunnel maintenance. However, existing mobile inspection methods still struggle to balance high-speed operation, fine-crack recognition, and comprehensive assessment of multiple defects. This study proposes an automatic recognition and quantitative assessment method for multiple visible defects in highway tunnels based on a vehicle-mounted multisensor inspection system. The system integrates high-resolution imaging, infrared illumination, 3D laser scanning, mileage positioning, and high-speed data storage, enabling continuous full-section data acquisition at speeds up to 80 km/h. A structural-feature-constrained mileage correction strategy is developed to reduce accumulated localization errors. For crack analysis, a multilevel framework combining two-stage CNN screening, cascaded segmentation, crack trajectory tracking, and subpixel edge extraction is established for crack recognition and 0.1 mm-level width measurement. Water leakage and spalling are extracted through visible–infrared image fusion and adaptive boundary refinement, while cross-sectional deformation is calculated using 3D tunnel axis reconstruction, point-cloud filtering, and cross-section fitting. Field tests and controlled experiments demonstrate that the system can rapidly identify, locate, and quantify multiple tunnel defects, providing a practical reference for intelligent tunnel inspection and maintenance.

## 1. Introduction

Highway tunnels are an essential component of transportation infrastructure. As a large number of tunnels have gradually entered the stage of long-term operation and maintenance, structural condition assessment and defect inspection have become increasingly important [1,2,3,4,5]. During construction and operation, tunnel linings are susceptible to visible defects such as cracks, water leakage, spalling, and deformation due to environmental actions and other adverse factors [6,7]. These defects not only reflect the local damage state of the lining structure, but are also closely related to tunnel durability and operational safety. Therefore, rapid, continuous, and accurate inspection of visible tunnel defects is fundamental to highway tunnel maintenance and structural safety assessment.

Conventional tunnel inspection has largely relied on manual visual surveys and measurements using handheld devices. Although these methods allow inspectors to assess defects based on field experience, they are often inefficient, subjective, and may interfere with normal traffic operation [8,9,10,11]. With the development of computer technologies, digital image processing methods, such as threshold segmentation and edge detection, have gradually been applied to tunnel defect recognition [12,13]. These methods enable automatic extraction and quantitative analysis of defect regions; however, their performance is usually sensitive to the quality of input images. In tunnel images, uneven illumination, noise, and non-defect textures can affect the extraction of defect features. As a result, high-quality defect images often need to be manually selected before further processing in practical applications [14]. In recent years, deep learning methods have improved recognition performance under complex backgrounds by automatically learning defect features, and have been increasingly used for tunnel defect detection [15,16,17]. Nevertheless, most existing studies have focused on a single type of defect, and the adopted models are commonly designed for specific tasks such as image classification, object detection, or semantic segmentation. Although some studies have attempted to combine multiple algorithms or fuse image and point cloud data, key procedures such as defect data acquisition, spatial localization, intelligent recognition, and quantitative assessment are still treated as relatively independent steps in many studies. Therefore, a complete inspection workflow for multiple types of tunnel defects remains to be further developed. Multisensor mobile inspection systems can provide complementary information for defect recognition, localization, and quantification by integrating visible-light images, infrared data, laser scanning, and mileage information. However, the synchronous acquisition and collaborative processing of multisource data, as well as the establishment of an integrated inspection workflow for multiple defect types, remain key challenges in automated tunnel inspection.

Considerable progress has been made in tunnel crack detection, image acquisition, and defect recognition. For instance, an early inspection system based on continuously scanned images verified the feasibility of continuous image acquisition for elongated facilities and automatic extraction of concrete cracks, providing a technical reference for subsequent mobile inspection of visible tunnel defects [18]. In recent years, deep learning methods based on object detection, semantic segmentation, and instance segmentation have been widely used for crack recognition and pixel-level extraction, achieving promising performance in complex backgrounds [19,20,21,22]. These methods have also been extended to reinforced concrete structures and post-earthquake damage assessment, demonstrating their potential for automated image-based crack detection under complex structural damage conditions [23]. Meanwhile, the development of multisource sensing technologies, such as 3D laser scanning, infrared thermography [24], inertial navigation, and mileage encoders, has provided new technical approaches for tunnel profile deformation calculation, water leakage recognition, and spatial localization of defects [25,26]. However, existing studies have mainly focused on single-defect recognition, local algorithm optimization, or low-speed inspection scenarios. Few efforts have established an integrated framework capable of simultaneously supporting high-speed data acquisition, defect localization, automatic recognition, and quantitative characterization of multiple tunnel defects. Moreover, the accuracy of defect recognition and measurement still requires further improvement.

The main performance characteristics of existing vehicle-mounted single-sensor and multisensor tunnel inspection systems are summarized in Table 1. Overall, current systems still involve trade-offs among inspection speed, imaging resolution, and defect coverage. Most mobile inspection systems operate at speeds below 50 km/h, making it difficult to continuously acquire full-section tunnel data under conditions close to normal traffic flow in highway tunnels. In addition, the minimum detectable crack widths reported in previous studies are mostly in the range of 0.2–0.5 mm, indicating that the recognition and quantification of fine cracks still require improvement. Existing studies have also tended to focus on crack detection, point-cloud-based deformation analysis, or a specific type of defect, while the coordinated integration of multisource data acquisition, synchronous localization, multi-defect recognition, and quantitative assessment remains insufficient. Therefore, the key challenge is not the lack of an individual sensor or recognition algorithm, but the absence of an integrated technical framework suitable for high-speed mobile tunnel inspection. To address this issue, this study develops a vehicle-mounted multisensor tunnel inspection system and establishes an integrated workflow for full-section data acquisition, spatial localization, crack recognition and width measurement, water leakage and spalling extraction, and cross-sectional deformation analysis. The proposed system can continuously acquire full-section tunnel data at speeds of up to 80 km/h and identify and quantify cracks with widths not less than 0.1 mm. It can also recognize and quantify water leakage and spalling regions, while tunnel cross-sectional deformation is calculated using 3D laser point clouds. Specifically, a multisensor mobile inspection platform is developed by integrating high-resolution image acquisition, 3D laser scanning, infrared illumination, mileage positioning, and high-throughput data storage modules. A structural-marker-constrained mileage correction method is proposed to reduce accumulated mileage errors and improve longitudinal defect localization. A multilevel image recognition framework is constructed for crack screening, recognition, and width measurement. Visible–infrared image fusion and adaptive boundary refinement are adopted to extract and quantify water leakage and spalling regions, and 3D point-cloud processing is used for tunnel cross-sectional deformation calculation. Finally, field tests in an operating highway tunnel in China are conducted to verify the feasibility and detection accuracy of the proposed system and methods.

**Figure 1:**
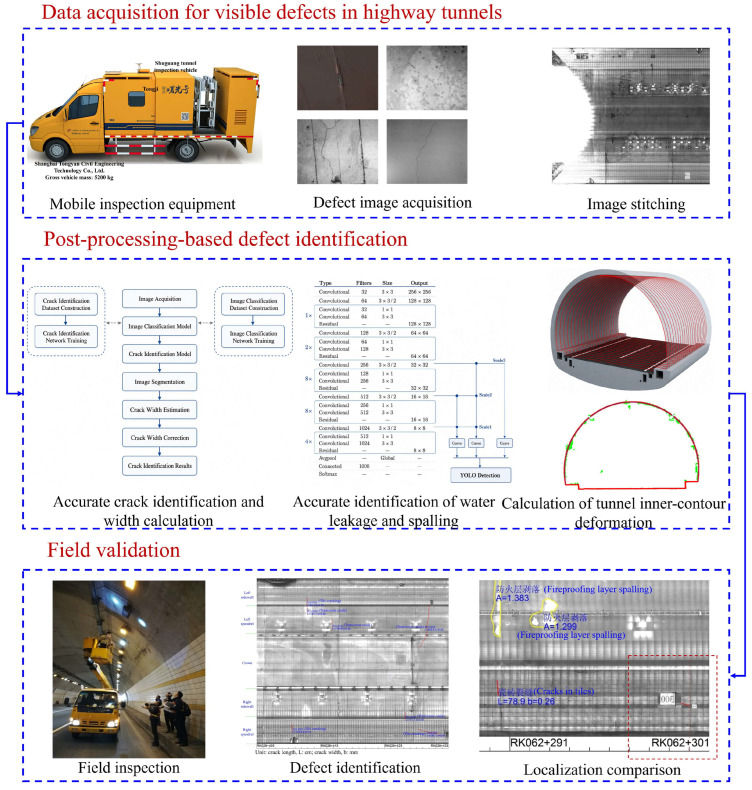
Research framework.

The research framework is shown in Figure 1. The remainder of this paper is organized as follows. Section 2 describes the overall design of the mobile inspection equipment and the working principles of its key subsystems. Section 3 presents the algorithms for defect localization, crack recognition, water leakage and spalling detection, and deformation calculation. Section 4 reports the field test procedure and results. Section 5 summarizes the main conclusions.

## 2. Design Parameters of the Multisensor Mobile Inspection Equipment

### 2.1. Overall Design

**Figure 2:**
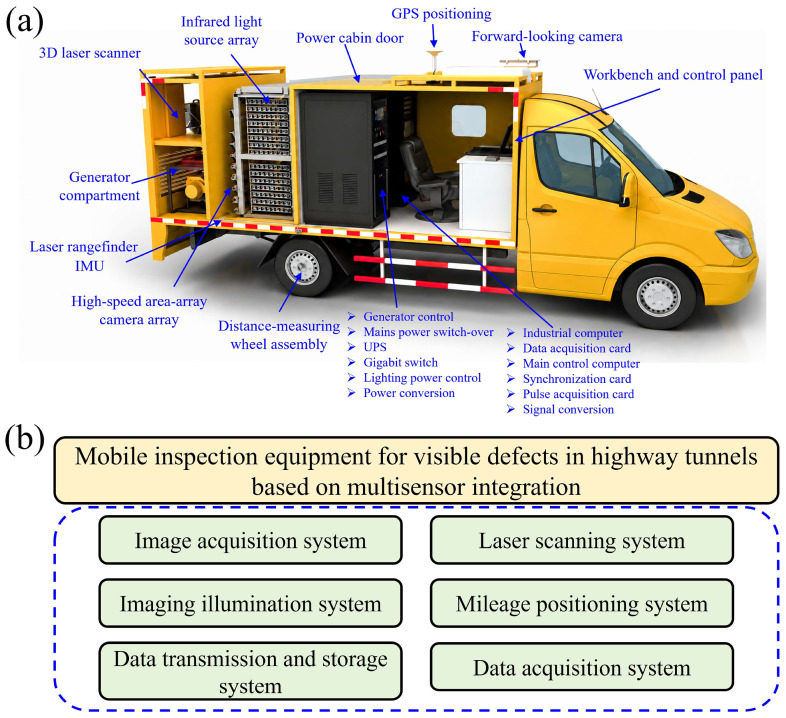
Composition of the mobile inspection equipment: (**a**) main components; (**b**) system modules.

Figure 2 shows the developed mobile inspection equipment for visible defects in highway tunnels. The equipment consists of a vehicle-mounted platform and a multisource acquisition system. The platform can be adapted from an appropriate chassis according to the scale and spatial layout of the acquisition system. The multisource acquisition system integrates image acquisition, 3D laser scanning, imaging illumination, data transmission and storage, mileage positioning, power supply, and inspection control modules. The image acquisition module, composed of multiple area-array CCD cameras, captures visible images of the tunnel lining. The 3D laser scanning module records the spatial profiles of the lining structure and auxiliary facilities. The imaging illumination module provides stable lighting to ensure image quality, while the data transmission and storage module supports real-time transmission and efficient storage of inspection data. The mileage positioning module determines the spatial position of the inspection vehicle inside the tunnel, providing the basis for defect localization and multi-period data comparison. The power supply and inspection control modules provide stable power and centralized control for the acquisition devices, respectively.

### 2.2. Image Acquisition System

**Figure 3:**
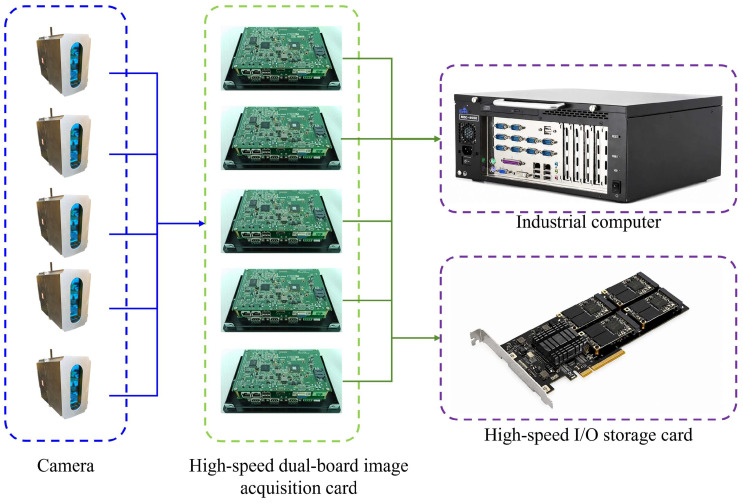
Architecture of the image acquisition and storage system.

As shown in Figure 3, the image acquisition system consists of industrial cameras, high-resolution lenses, infrared illumination arrays, data transmission cables, and power cables. The camera configuration was determined by considering the cross-sectional dimensions of typical two-lane highway tunnels, the travelling position of the inspection vehicle, the available mounting space, the lens field of view, the working distance, and the target spatial resolution. Thirty CCD industrial cameras were arranged circumferentially on the inspection vehicle to form a continuous imaging array. Because the projected field of view and spatial resolution of a single camera on the lining surface vary with imaging distance and viewing angle, the camera positions and viewing directions were designed to ensure continuous coverage of the tunnel lining, interior panels, suspended ceiling structures, pavement, and drainage areas during a single pass of the inspection vehicle. The designed full-section spatial resolution of the system is approximately 0.2 mm/pixel, which represents a balance among fine-defect imaging capability, surface coverage, and the large data volume generated during high-speed inspection. In local near-field regions, where the imaging distance is shorter and the object-side projection area is smaller, the spatial resolution can reach approximately 0.1 mm/pixel. Combined with subsequent pixel-level crack segmentation, subpixel boundary localization, and width correction, this near-field resolution provides the image basis for identifying cracks at the 0.1 mm level. The imaging centers of all cameras were arranged within the same tunnel cross-sectional plane and operated using synchronized triggering. This configuration reduces longitudinal misalignment among images acquired by different cameras under vehicle motion and improves the consistency of full-section image stitching. Although increasing the number of cameras can enhance imaging redundancy, it also increases calibration and synchronization difficulty as well as system cost. Therefore, the configuration of 30 cameras was determined by comprehensively balancing full-section coverage, imaging quality, and engineering feasibility. The acquired images are synchronously transmitted and stored using an industrial computer, image acquisition cards, and high-speed solid-state drives, ensuring stable acquisition of large-scale image data. To ensure synchronized acquisition of multisource data during high-speed inspection, the system adopts a hardware-triggered synchronization scheme controlled by a mileage encoder and an FPGA module. Encoder pulses are used as a unified mileage-based trigger source. After signal conditioning, frequency division, and distribution by the FPGA synchronization control module, the trigger signals are sent to the camera array and related acquisition modules, thereby enabling equal-distance data acquisition and synchronous exposure of multiple cameras. At an inspection speed of 80 km/h, the 30 CCD cameras can perform synchronized imaging and data acquisition, and the maximum image spatial misalignment caused by trigger synchronization errors is controlled within 1 mm.

Before field inspection, each CCD camera was calibrated and adjusted to ensure stable imaging performance. The calibration procedure included lens focusing, distortion correction, matching between the camera field of view and the illuminated region, and verification of the pixel-to-physical-size conversion. Calibration targets with known dimensions were used to validate the imaging scale and ensure that the designed imaging resolution satisfied the requirements for crack recognition and quantification. After calibration, the cameras, lenses, and mounting structures were mechanically locked to maintain imaging stability during high-speed inspection. To ensure image quality, the cameras were operated with fixed exposure time and fixed gain settings. Before field inspection, the upper limit of the exposure time was determined according to the maximum inspection speed and the designed imaging resolution, so that motion blur during exposure was limited to approximately two pixels. The lens aperture, camera gain, and infrared illumination intensity were then adjusted to ensure clear imaging of lining textures and fine cracks while avoiding overexposure. During continuous data acquisition, these parameters were kept unchanged to reduce brightness fluctuations caused by automatic exposure adjustment. Multi-camera image stitching errors are mainly affected by camera calibration, field-of-view overlap, and trigger synchronization. In this study, the stitching error was controlled through camera imaging calibration, mileage-encoder-based synchronous triggering, and field-of-view matching between adjacent cameras, thereby ensuring the continuity of full-section tunnel images and the consistency of defect localization.

### 2.3. Laser Scanning System

**Figure 4:**
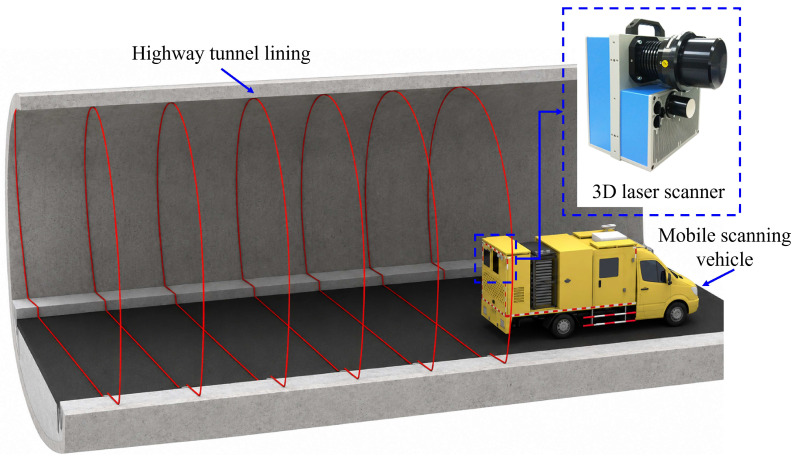
Schematic diagram of the 3D laser scanning system for tunnel lining measurement.

The laser scanning module operates in a pixel-by-pixel scanning mode, with a point cloud acquisition rate exceeding 1 million points/s and a cross-sectional scanning speed of up to 200 r/s. This enables high-density and high-precision scanning of closely spaced tunnel cross-sections. As shown in Figure 4, the laser scanner is mounted on the mobile inspection vehicle and operates in line-scanning mode as the platform moves forward. During inspection, the laser beam remains perpendicular to the travelling direction of the inspection platform. The laser emitter rotates 360° around its axis and emits high-frequency laser beams toward the target surface. The sensor receives the signals reflected from the inner surface of the tunnel lining and records the corresponding time or phase differences. By analyzing the intensity and phase information of the emitted and reflected signals, point cloud data of the lining surface can be obtained. Tunnel cross-sectional point clouds were acquired using a Z+F PROFILER® 9012 laser scanner (Zoller + Fröhlich GmbH, Wangen im Allgäu, Germany). According to the technical specifications of the instrument, the measurement error of the PROFILER 9012 is affected by target reflectivity and scanning distance. Within the effective measurement range of 4–10 m commonly used in tunnel inspection, the intrinsic measurement error of the scanner for dark-grey targets is approximately 0.3 mm.

The point cloud data are further synchronized and spatially calibrated with the mileage information along the tunnel axis recorded by the mileage positioning system. Multiple cross-sectional point clouds are then stitched to generate a complete 3D point cloud dataset. As the inspection vehicle travels along the tunnel axis, the rotation axis of the scanner remains approximately parallel to the tangent direction of the tunnel axis. This configuration makes the laser incidence direction close to the normal direction of the tunnel wall, improving the spatial consistency of the point cloud data and the accuracy of profile deformation calculation.

### 2.4. Imaging Illumination System

The lighting conditions in operating highway tunnels are generally poor; therefore, active illumination is required to obtain clear and stable lining images. Because visible-light illumination may interfere with traffic operation, an invisible infrared illumination scheme was adopted. Based on the internal profile of a two-lane tunnel and the camera imaging distance, the near-field and far-field illumination distances were approximately 3.5 m and 7.5 m, respectively. The illuminated surfaces mainly included concrete linings, ceramic tiles, and fireproof coatings. To meet the imaging requirements under high-speed inspection, the illumination system was designed to maintain a mean grayscale value greater than 50 in the acquired images, while ensuring good brightness uniformity.

**Figure 5:**
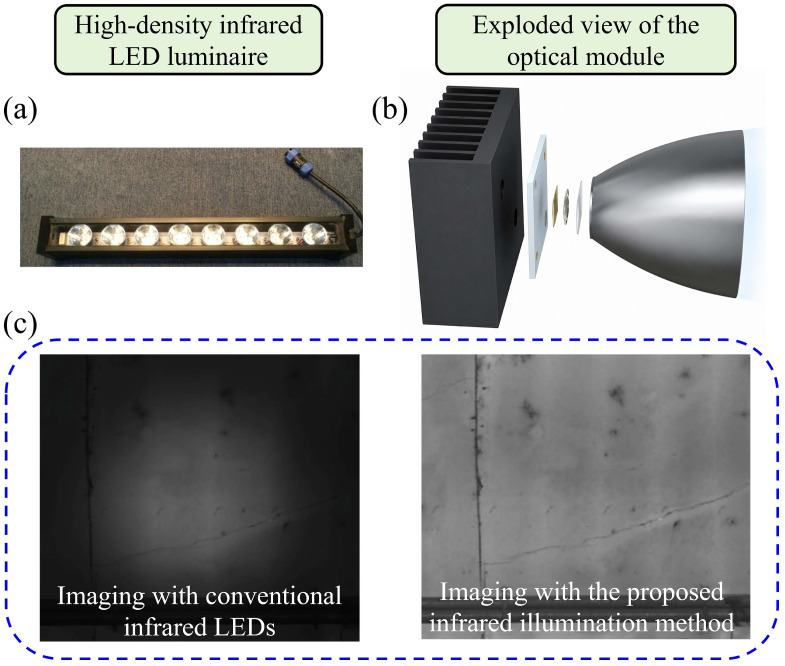
Imaging illumination system: (**a**) high-density infrared LED luminaire; (**b**) structure of the illumination device; (**c**) performance comparison.

Conventional single-chip infrared LEDs typically have an output power below 0.2 W, which is insufficient for long-distance imaging. Multi-chip integrated sources can provide higher power, but are often limited by heat dissipation and reduced service life. To address these limitations, a high-density infrared illumination device was developed using a high-thermal-conductivity substrate, chip-level die-bonding soldering, and fully inorganic packaging. Nine infrared LED chips were integrated into a 4 mm × 4 mm emitting array, and a sapphire window was used to improve optical efficiency and stability. The light source cavity and heat dissipation structure were integrated through an aluminum extrusion process, and a high-voltage direct-current driving technique was adopted to reduce thermal resistance. Compared with conventional infrared LED sources, the developed device achieved an optical power of 3 W and a thermal resistance of 2.5 K/W within a 20 mm^2^ emitting area, increasing the output power by approximately 15 times and reducing the thermal resistance by about 75%. The device provided a long-distance imaging brightness uniformity of up to 95%, satisfying the requirements for full-section image acquisition at an inspection speed of 80 km/h. As shown in Figure 5, the high-density infrared illumination system produced clearer images and more uniform lighting than conventional infrared LED illumination under the same imaging conditions.

### 2.5. Positioning, Data Acquisition and Storage System

The positioning and data acquisition system is based on time synchronization and integrates a distance-measuring wheel, inertial navigation, laser rangefinders, GPS, 3D laser scanners, and feature-marker recognition modules to achieve multisource cooperative positioning and spatial registration of inspection data. The distance-measuring wheel is used for mileage positioning, the inertial navigation system records vehicle attitude, the laser rangefinders control the relative distance between the vehicle and the tunnel sidewall, GPS records the entrance and exit positions of the tunnel, and feature-marker recognition is used for reverse mileage correction. The data acquisition system was developed on the LabVIEW platform and can synchronously acquire and store vehicle attitude, mileage positioning, lining images, video images, 3D point clouds, infrared thermal images, environmental parameters, and equipment status data. During inspection, the image data rate is approximately 600 MB/s, and the data volume exceeds 10 GB per kilometer. To meet the requirements of high-throughput data transmission and storage, the system adopts DMA1800 high-speed data transmission technology and is equipped with a PCIe ×4 Gen2 Camera Link acquisition card and five 1 TB high-speed SSDs, which can support an inspection capacity of 300 km/d.

## 3. Key Techniques for Defect Recognition

### 3.1. Longitudinal Positioning Method Inside Tunnels

Inside tunnels, satellite signals are usually unavailable; therefore, defect localization relies on vehicle-mounted multisource sensing. Circumferential positioning is achieved by mapping defect pixels to the tunnel cross-sectional coordinate system using 3D laser scanning data, camera installation parameters, and field-of-view information. In contrast, longitudinal positioning is more affected by wheel slip, time synchronization errors, and accumulated mileage errors, making mileage correction essential for accurate defect localization.

A multilevel longitudinal positioning method combining encoder-based preliminary positioning and image-feature-based calibration was adopted. A synchronization control card establishes a unified time sequence among the laser scanner, incremental encoder, and camera array, enabling synchronous acquisition of multisource data. The encoder provides preliminary mileage information for the inspection vehicle, images, and point clouds. To reduce accumulated errors during long-distance inspection, construction joints are used as calibration references because they are periodically distributed along the tunnel, have stable positions, and exhibit distinct grayscale variations in unfolded lining images. By matching construction joint image features with acquisition time and actual mileage, the encoder mileage can be reversely corrected.

**Figure 6:**
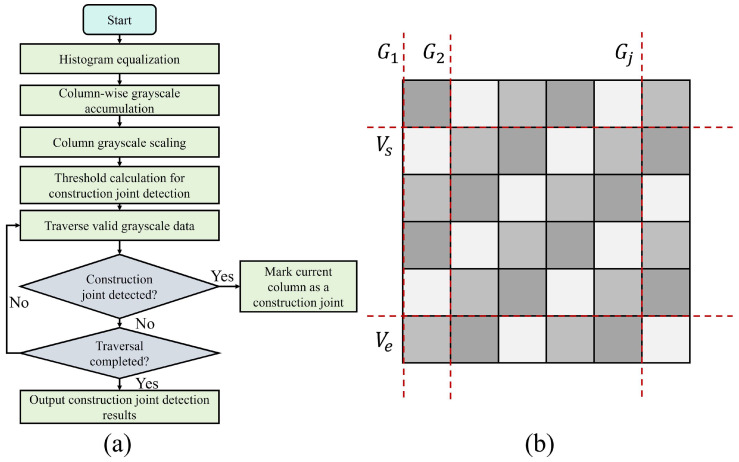
Construction joint recognition based on grayscale accumulation: (**a**) recognition flowchart; (**b**) schematic of grayscale accumulation.

Figure 6a shows the construction joint recognition procedure. The two-dimensional grayscale unfolded image of the tunnel lining is first preprocessed. Because construction joints generally appear as dark linear features extending along the circumferential direction, they cause significant column-wise grayscale variations at their longitudinal positions. Therefore, candidate joint positions are extracted using column-wise grayscale accumulation. As shown in Figure 6b, the grayscale accumulation value of the j-th column, $G_{j}$, is calculated within the statistical range from $V_{s}$ to $V_{e}$ as follows:

(1) $$G_{j}=\sum_{i=V_{s}}^{V_{e}}g\left(i,j\right),$$

where g(i,j) is the grayscale value of the pixel located in the i-th row and j-th column. To improve comparability among different images, the accumulated grayscale values are scaled and denoted as $G_{j}^{\prime }$. The construction joint detection threshold $T_{d}$ is then determined from the scaled grayscale sequence:

(2) $$T_{d}=VR_{d},$$

(3) $$V=G_{max}^{\prime }-G_{min}^{\prime },$$

(4) $$G_{max}^{\prime }=\max\left(G_{j}^{\prime }|j\epsilon \left[1,W\right]\right),$$

(5) $$G_{min}^{\prime }=\min\left(G_{j}^{\prime }|j\epsilon \left[1,W\right]\right),$$

where V is the scaled grayscale difference, $R_{d}$ is the discrimination coefficient for construction joint detection and is set to 0.5 in this study, and $G_{max}$ and $G_{min}$ are the maximum and minimum values of the scaled column-wise grayscale accumulation sequence, respectively. After $T_{d}$ is obtained, all valid columns are traversed. For each valid column j, the mean grayscale accumulation values of N sampled columns on its left and right sides are calculated as $G_{l}^{\prime }$ and $G_{r}^{\prime }$:

(6) $$G_{l}^{\prime }=\frac{1}{N}\sum_{m=j-S_{l}-N}^{j-S_{l}}G_{m}^{\prime },$$

(7) $$G_{r}^{\prime }=\frac{1}{N}\sum_{m=j+S_{r}}^{j+S_{r}+N}G_{m}^{\prime },$$

where $S_{l}$ and $S_{r}$ are the offsets of the left and right sampled regions from the current column, respectively, and N is the number of sampled columns. The grayscale differences between the current column and its left and right neighborhoods are then calculated as:

(8) $$D_{l}=G_{l}^{\prime }-G_{j}^{\prime },$$

(9) $$D_{r}=G_{r}^{\prime }-G_{j}^{\prime },$$

where $D_{l}$ and $D_{r}$ denote the grayscale differences between the current column and the left and right sampled regions, respectively. If both $D_{l}$ and $D_{r}$ exceed $T_{d}$, the current column is identified as a construction joint; otherwise, it is regarded as a non-joint feature caused by local texture, shadows, or noise.

After construction joints are identified, their image acquisition time, corresponding laser scanning data, and encoder-based preliminary mileage are back-calculated from the multisource synchronization sequence. The actual mileage is obtained from the predefined structural feature mileage database. The identified construction joints are then used as control points to reversely correct the encoder mileage sequence, and the mileage between adjacent control points is uniformly calibrated, thereby reducing accumulated errors and improving longitudinal defect localization accuracy.

### 3.2. Crack Recognition and Width Calculation Method

**Figure 7:**
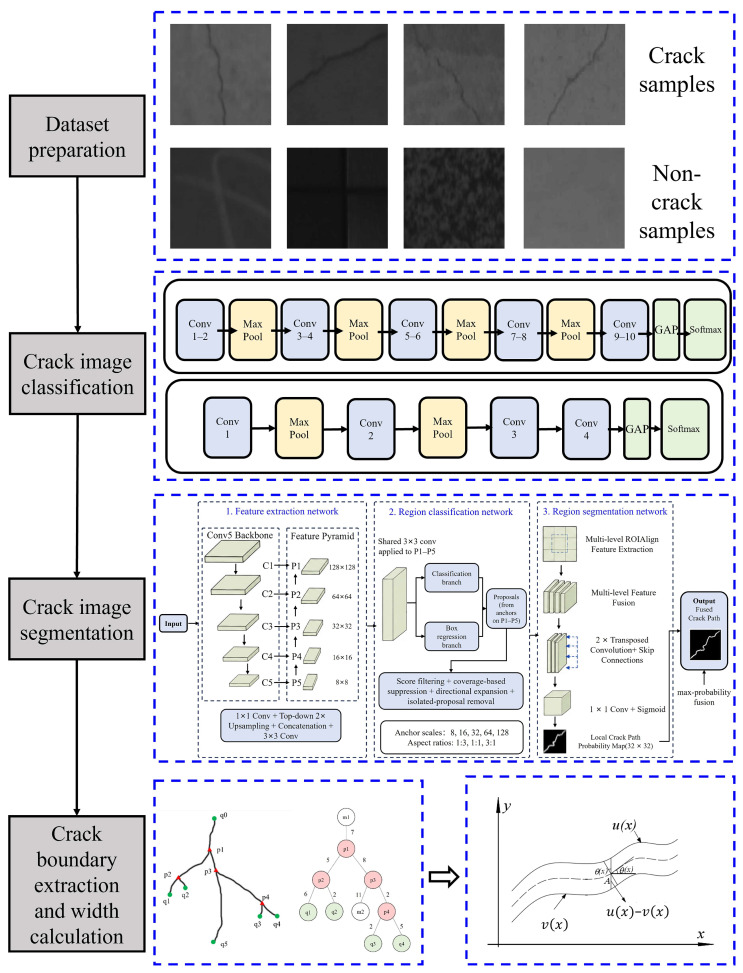
Multilevel framework for crack recognition and width calculation.

For tunnel lining cracks under complex backgrounds and various interference conditions, an automatic crack recognition framework combining deep image classification and object segmentation was developed, as shown in Figure 7.

Visible-light images of tunnel linings were collected from multiple highway tunnels in 16 provinces and municipalities in China, resulting in more than 300 million raw images with a resolution of 2560 × 2048 pixels. After manual screening, annotation, and review, 3 million images were retained for crack classification, with a 1:1 ratio of crack-positive samples to crack-free negative samples. In addition, 500,000 original image patches were prepared for crack segmentation, with equal numbers of positive and negative samples. For positive samples, annotators manually traced the crack paths and generated corresponding line-shaped binary labels, whereas negative samples were assigned full-background labels after manual confirmation. A three-level quality control procedure was adopted for data annotation. The annotation team consisted of 38 annotators with a civil engineering background and at least a bachelor’s degree, approximately 11 engineers with relevant engineering experience, and 3 industry experts. The classification dataset was divided into training, validation, and test sets at a ratio of 8:1:1, while the segmentation dataset was divided into training and validation sets at a ratio of 9:1. Data augmentation was applied only to the segmentation training set after dataset splitting, and the validation set was not augmented. Random rotation, brightness adjustment, and Mosaic augmentation were used during training, producing approximately 5 million training instances. Images and patches cropped or generated from the same defect instance were assigned to the same subset to avoid overlap of homologous samples across different subsets. The dataset was split at the image level rather than the tunnel level; therefore, the reported results mainly reflect model performance under a mixed multi-tunnel data distribution.

A lightweight two-stage convolutional neural network (CNN) framework was adopted for crack image screening and classification. The first-stage CNN takes tunnel lining images resized to 512 × 512 pixels as input and is used for preliminary screening of images that may contain cracks. Subsequently, a 256 × 256-pixel sliding window is applied to the suspected crack images for local scanning, and the resulting image patches are fed into the second-stage CNN to further identify crack regions. Both networks were trained using stochastic gradient descent (SGD), with an initial learning rate of 0.001, a momentum of 0.9, a batch size of 64, and a maximum of 2000 training epochs. An early-stopping strategy was adopted, and categorical cross-entropy was used as the loss function. Considering that the classification dataset contains 3 million real tunnel images collected from diverse sources, no additional data augmentation was applied during classification network training. The predicted class was determined according to the maximum Softmax output probability, without an additional manually defined classification threshold. The total parameter storage size of the two-stage CNN is approximately 4.2 MB. The detailed network architecture and training parameters are summarized in Table 2.

To achieve pixel-level segmentation of crack paths, a cascaded network consisting of multiscale feature extraction, candidate-region classification, and region segmentation modules was constructed. The input to the network was the 256 × 256-pixel suspected crack patches selected by the two-stage CNN, and the network architecture and main parameters are summarized in Table 3. The multiscale feature extraction module consists of a five-level convolutional backbone and a feature pyramid. The backbone extracts features at different spatial scales through progressive downsampling. Features at each level are first projected to a unified channel dimension using 1 × 1 convolutions. The deep features are then upsampled by a factor of two in a top-down manner and concatenated with the corresponding shallow features along the channel dimension. The concatenated features are further processed using 3 × 3 convolutions to adjust the channel dimension, thereby integrating shallow edge-texture information with deep semantic information. The candidate-region classification module consists of shared convolutional layers, a classification branch, and a bounding-box regression branch. Multiscale anchors with different aspect ratios are generated on the pyramid feature maps. The classification branch predicts the probability that each anchor contains a crack, while the regression branch refines the position and size of the anchor. During training, the manually traced linear crack labels are automatically converted into local ground-truth boxes, and positive and negative samples are assigned according to the intersection-over-union between anchors and ground-truth boxes. During inference, low-quality and duplicate candidate boxes are removed based on confidence scores and overlap coverage. Directional dilation and isolated-box filtering are further applied to improve the continuity of candidate crack regions. The overlap coverage ratio is defined as the ratio of the intersection area of two candidate boxes to the area of the smaller candidate box. The region segmentation module consists of multilayer region feature extraction, progressive upsampling fusion, and a pixel classification layer. Candidate regions are assigned to the corresponding pyramid feature levels according to their scales, and ROIAlign is used to extract fixed-size region features from the assigned level and subsequent deeper feature maps. The deep region features are progressively upsampled through transposed convolutions and concatenated with adjacent shallow features through skip connections. Finally, a 1 × 1 convolution followed by a sigmoid function is used to generate the local crack-path probability map. The local segmentation results are mapped back to the input image coordinate system according to the candidate-box positions, and overlapping regions are fused using the maximum probability, thereby obtaining a complete linear crack path for subsequent subpixel edge extraction and crack-width calculation. The model contains approximately 5.6 million trainable parameters, and the weight file size is approximately 22.4 MB. The total loss of the model consists of the candidate-region classification loss, bounding-box regression loss, and crack-path segmentation loss:

(10) $$L_{total}=L_{cls}+L_{box}+L_{seg},$$

where $L_{cls}$ is the binary cross-entropy loss used to determine whether a candidate anchor contains a crack, and $L_{box}$ is the Smooth L1 loss applied only to positive anchors to refine the position and size of candidate boxes. Considering that crack pixels account for a much smaller proportion than background pixels, the segmentation branch adopts a combination of weighted binary cross-entropy loss and Dice loss:

(11) $$L_{seg}=0.5L_{WBCE}+0.5L_{Dice},$$

where $L_{WBCE}$ is used to reduce the influence of the imbalance between crack and background pixels, and $L_{Dice}$ is used to improve the overlap between the predicted crack regions and the ground-truth annotations. The losses of the three branches are jointly optimized with equal weights.

For the classification model, Precision, Recall, and F1 were used as evaluation metrics. Here, TP denotes the number of crack image samples correctly identified as cracks, FP denotes the number of crack-free image samples incorrectly classified as cracks, and FN denotes the number of crack image samples that were not detected. These metrics are defined as follows:

(12) $$Precision=\frac{TP}{TP+FP},$$

(13) $$Recall=\frac{TP}{TP+FN},$$

(14) $$F1=\frac{2TP}{2TP+FP+FN}.$$

For the crack-path segmentation model, pixel-level Recall, IoU, and Dice were adopted for evaluation. In this case, TP denotes the number of crack pixels correctly identified, FP denotes the number of background pixels incorrectly classified as crack pixels, and FN denotes the number of crack pixels that were not detected. Pixel-level Recall was calculated using the same form as Equation (13), while IoU and Dice are defined as follows:

(15) $$IoU=\frac{TP}{TP+FP+FN},$$

(16) $$Dice=\frac{2TP}{2TP+FP+FN}.$$

To further improve crack characterization, crack trajectory tracking and subpixel edge extraction were performed based on the segmentation results. A continuous crack centerline was first obtained through trajectory tracking and branch pruning on the binary crack image. Subsequently, a Zernike-moment-based subpixel edge detection method was employed to accurately extract crack boundaries. By combining the crack centerline and boundary information, a dynamic radial calculation model was established for crack morphology characterization and width measurement, thereby improving crack recognition completeness and localization accuracy under complex background conditions.

As shown in Figure 7, crack width was further calculated based on the pixel-level segmentation results. For cracks with relatively continuous boundaries, the upper and lower crack boundaries can be represented by two continuous functions, u(x) and v(x), respectively. The crack centerline C(x) can be expressed as:

(17) $$C\left(x\right)=\frac{u\left(x\right)+v\left(x\right)}{2}.$$

The crack width depends not only on the pixel distance between the two boundaries but also on the local inclination angle of the crack centerline. Therefore, the crack width at position x can be expressed as:

(18) $$W\left(x\right)=P\left(x\right)cos\theta \left(x\right),$$

where P(x)=u(x)−v(x) represents the apparent crack width measured along the image coordinate direction, and θ(x) denotes the local inclination angle of the crack centerline at position x.

For vertical and inclined cracks, the crack width can be calculated based on the distribution of crack pixels in each row of the binary crack image. Suppose that the binary crack image contains m rows and n columns. Let P(i) denote the number of crack pixels in the i-th row and Z(i,k) denote the column coordinate of the k-th crack pixel. The center coordinate of the crack in the i-th row can be expressed as:

(19) $$M\left(i\right)=\frac{\sum_{k=1}^{P\left(i\right)}Z\left(i,k\right)}{P\left(i\right)},$$

where M(i) is the column coordinate of the crack center point in the i-th row. The crack centerline is obtained by connecting the center points of adjacent rows. The local inclination angle of the crack centerline at the i-th row is then calculated as:

(20) $$\theta_{i}=\arctan\left[\frac{M\left(i+1\right)-M\left(i-1\right)}{2}\right],$$

where $\theta_{i}$ denotes the local inclination angle of the crack centerline at the i-th row. The actual crack width at the i-th row can be expressed as:

(21) $$\omega_{i}=\eta P\left(i\right)cos\theta \left(i\right),$$

where $\omega_{i}$ is the crack width at the i-th row and η is the image pixel resolution.

For transverse cracks and cracks with large inclination angles, the same procedure can be applied in the column direction. The crack pixels in each column are counted to determine the corresponding crack center points, which are then connected to form the crack centerline. The crack width is subsequently corrected according to the local inclination angle of the centerline.

### 3.3. Water Leakage and Spalling Recognition

**Figure 8:**
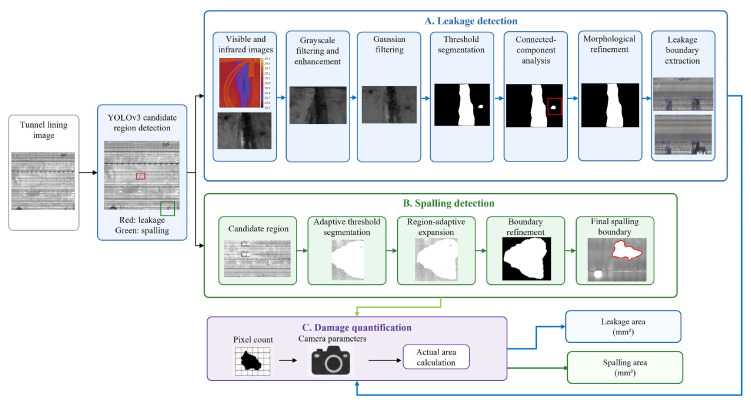
Recognition workflow for water leakage and spalling.

For the two typical visible defects of water leakage and spalling, this study adopted a recognition framework combining YOLOv3-based candidate region detection and image-processing-based boundary refinement, as shown in Figure 8. First, YOLOv3 was used to rapidly detect the entire tunnel lining image and locate candidate regions of suspected water leakage and spalling, thereby reducing the influence of complex backgrounds on subsequent boundary extraction. For water leakage detection, visible-light images and infrared thermal images were jointly used. Visible-light images provide texture and color information of the lining surface, whereas infrared thermal images reflect the temperature difference between leakage regions and dry surrounding areas. The candidate regions were then subjected to grayscale conversion, filtering, and enhancement to reduce the effects of uneven illumination and random noise. Gaussian filtering was adopted for image smoothing, and its kernel function is given by:

(22) $$G\left(x,y\right)=\frac{1}{2\pi \sigma^{2}}e^{-\frac{x^{2}+y^{2}}{2\sigma^{2}}},$$

where G(x,y) is the Gaussian kernel function, x and y are the pixel coordinates relative to the center of the filtering kernel, and σ is the standard deviation of the Gaussian kernel. Based on the enhanced images, threshold segmentation and connected component analysis were performed to extract candidate water leakage regions. Morphological processing was then applied to refine the region boundaries, remove isolated noise points, and smooth the leakage contours.

For spalling detection, a region-adaptive threshold segmentation method was applied within the detected candidate regions. Let $n_{i}$ be the number of pixels corresponding to the i-th grayscale level in the grayscale image, and n be the total number of pixels. The probability of the i-th grayscale level is expressed as:

(23) $$p_{i}=\frac{n_{i}}{n},$$

where $p_{i}$ is the probability of the i-th grayscale level, $n_{i}$ is the number of pixels at this grayscale level, and n is the total number of pixels in the image. After the optimal segmentation threshold was determined from the grayscale histogram, binary segmentation was performed to extract the spalling region. Considering that the initial segmentation result may contain incomplete boundaries or local missing areas, region growing was further used to compensate for boundary discontinuities and obtain a complete spalling region.

Finally, the actual defect area was calculated using the number of pixels in the extracted region and the camera imaging parameters. Let num be the number of pixels in the extracted defect region, $N_{num}$ be the total number of pixels in the whole image, h×w be the camera sensor size, d be the imaging distance, and f be the focal length. The actual area of the defect region can be expressed as:

(24) $$area=hw{\left(\frac{d}{f}\right)}^{2}\frac{num}{N_{num}}.$$

It should be noted that the area conversion in Equation (24) is not directly based on an uncorrected ideal camera model. Instead, it is performed after camera imaging calibration, distortion correction, and verification of the pixel-to-physical-size conversion. For the local field of view of a single camera, the tunnel lining surface is approximated as a local tangent plane. The imaging distance d is determined according to the camera installation geometry and is further verified on site using targets with known dimensions. The regions captured by different cameras are calculated using their respective calibrated imaging scales, thereby reducing the influence of camera arrangement, imaging distance, and tunnel curvature on area conversion.

### 3.4. Tunnel Profile Deformation Calculation

**Figure 9:**
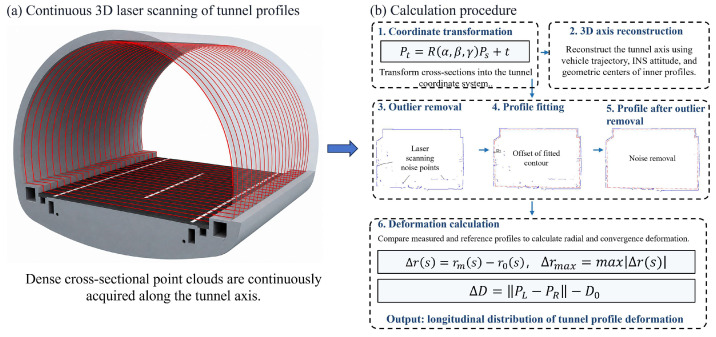
Tunnel deformation calculation method based on laser scanning.

A tunnel cross-sectional profile calculation method was established based on vehicle-mounted 3D laser scanning data, as shown in Figure 9. The motion state of the inspection vehicle and the cross-sectional point cloud data were obtained using the mileage positioning system, inertial navigation system, and 3D laser scanner. The scanned points were then transformed into the tunnel coordinate system. Let $P_{s}=(x_{s},y_{s},z_{s}{)}^{T}$ be the coordinate of a point in the scanner coordinate system, α, β, and γ be the vehicle attitude angles, and $t=(x_{0},y_{0},z_{0}{)}^{T}$ be the translation vector. The coordinate of this point in the tunnel coordinate system can be expressed as:

(25) $$P_{t}=R\left(\alpha ,\beta ,\gamma \right)P_{s}+t,$$

where $P_{t}$ is the transformed point coordinate in the tunnel coordinate system, R(α,β,γ) is the rotation matrix determined by the vehicle attitude angles, and t is the translation vector from the scanner coordinate system to the tunnel coordinate system. Through this transformation, cross-sectional point clouds acquired at different times can be unified within the same spatial reference frame.

On this basis, the tunnel 3D axis was reconstructed by integrating the vehicle trajectory, inertial navigation attitude data, and the inner-profile point cloud of the tunnel. The inertial navigation data were used to correct attitude variations during vehicle movement, and the geometric centers of continuously scanned inner profiles were extracted as axis control points along the tunnel longitudinal direction. A curve was then fitted to these control points to obtain the tunnel 3D axis. Taking this axis as the reference, local cross-sectional coordinate systems orthogonal to the tunnel axis were established, and the point clouds were projected onto the corresponding cross-sectional planes for continuous tunnel profile extraction.

The original point cloud may contain outliers unrelated to the tunnel profile because of interference from auxiliary facilities. To improve profile fitting accuracy, the point cloud was first mapped onto the cross-sectional plane and preliminarily partitioned according to the tunnel cross-sectional geometry and scanning configuration. Outliers were then removed by combining a distance threshold with least-squares fitting. Let $\Delta L_{i}$ be the distance from the i-th scanned point to the current fitted curve, and $L_{r}$ be the distance threshold. The outlier discrimination criterion is expressed as:

(26) $${Flag}_{i}=\left\{\begin{array}{c} 1,∆L_{i}>L_{r}\\ 0,∆L_{i}\leq L_{r} \end{array}\right.,$$

where $Flag_{i}=1$ indicates that the point is identified as an outlier and removed, whereas $Flag_{i}=0$ indicates that the point is retained as a valid profile point. Curve fitting and outlier removal were iteratively performed until the convergence criterion was satisfied, thereby obtaining a stable point set for the tunnel cross-sectional profile.

Least-squares fitting was then applied to the valid cross-sectional point cloud for profile reconstruction. Let $P_{i}$ denote the i-th valid point and C denote the fitted profile curve. The optimal fitted curve $C^{*}$ can be expressed as:

(27) $$C^{*}=arg\underset{C}{min}\sum_{i=1}^{N}d^{2}(P_{i},C),$$

where N is the number of valid points, and $d(P_{i},C)$ is the distance from point $P_{i}$ to the fitted curve C. This equation indicates that the optimal profile curve is obtained by minimizing the sum of squared distances between the point cloud and the fitted curve.

The measured profile was then compared with the design profile or a reference profile to calculate radial deformation. Let $r_{m}(s)$ be the measured radius or normal distance at position s, and $r_{0}(s)$ be the corresponding value of the reference profile. The profile deformation at position s can be expressed as:

(28) $$∆r\left(s\right)=r_{m}\left(s\right)-r_{0}\left(s\right).$$

The maximum radial deformation of the cross-section is then given by:

(29) $$∆r_{max}=max\left|∆r(s)\right|.$$

For cross-sections where convergence deformation is used as the evaluation indicator, symmetric control points on the left and right sidewalls or haunches can be selected to calculate the change in cross-sectional width relative to the reference profile. Let $P_{L}$ and $P_{R}$ denote the coordinates of the left and right control points in the measured cross-section, respectively, and let $D_{0}$ denote the corresponding width of the reference cross-section. The convergence deformation of the current cross-section can be expressed as:

(30) $$∆D=\left\|P_{L}-P_{R}\right\|-D_{0},$$

where ΔD is the convergence deformation, and $∥P_{L}-P_{R}∥$ is the distance between the left and right control points in the measured cross-section. By continuously extracting cross-sectional profiles along the tunnel and calculating Δr(s) or ΔD, the longitudinal distribution of tunnel profile deformation can be obtained.

## 4. Accuracy Validation

### 4.1. Test Method

**Figure 10:**
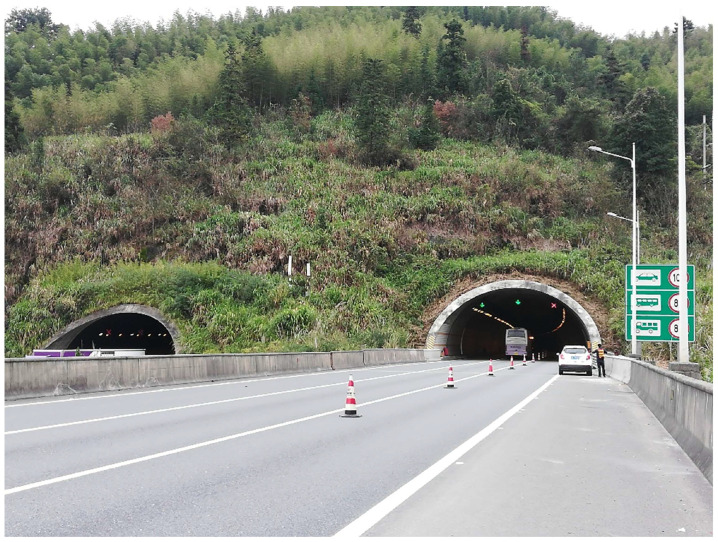
Tunnel site in Guangdong Province, China.

**Figure 11:**
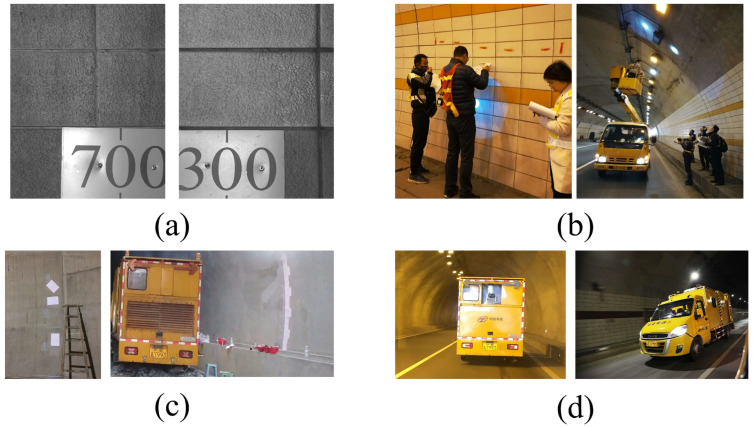
Field inspection: (**a**) hundred-meter marker; (**b**) crack detection; (**c**) spalling and water leakage detection; (**d**) deformation measurement.

As shown in Figure 10 and Figure 11, field tests were conducted in a tunnel in Guangdong Province, China. The tests included longitudinal defect positioning, crack recognition, water leakage and spalling recognition, and tunnel profile deformation measurement.

For the longitudinal positioning test, hundred-meter markers were fixed to the tunnel sidewall using expansion bolts. The inspection equipment was then operated to acquire tunnel data, and the mileage identified by the system was compared with the actual mileage. For the crack recognition test, 408 cracks on the inner surface of the tunnel were inspected using both manual inspection and the mobile inspection equipment. For the water leakage and spalling tests, A4 paper sheets with known sizes were attached to the tunnel surface as controlled targets to evaluate the boundary extraction and area calculation accuracy of the inspection equipment. In addition, representative real water leakage and spalling cases were used to examine the applicability of the proposed method under actual tunnel surface conditions. For the profile deformation test, a selected tunnel cross-section was measured using both a total station and the inspection equipment, and the obtained profiles were compared.

### 4.2. Results

#### 4.2.1. Defect Positioning Test

**Figure 12:**
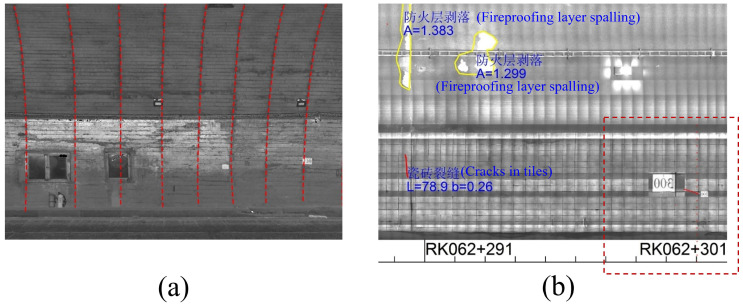
Two-dimensional mileage-expanded map obtained by the tunnel inspection equipment: (**a**) construction joint recognition; (**b**) mileage positioning.

The original images were stitched using encoder data and hundred-meter markers to generate a two-dimensional expanded map for visualizing defect recognition results inside the tunnel, as shown in Figure 12. The inspection equipment accurately identified the construction joints and determined the mileage position of RK062+300, which corresponded to the 300 m marker. The longitudinal positioning error was less than 1 cm, confirming the effectiveness of the proposed method for defect localization.

#### 4.2.2. Crack Recognition Test

**Figure 13:**
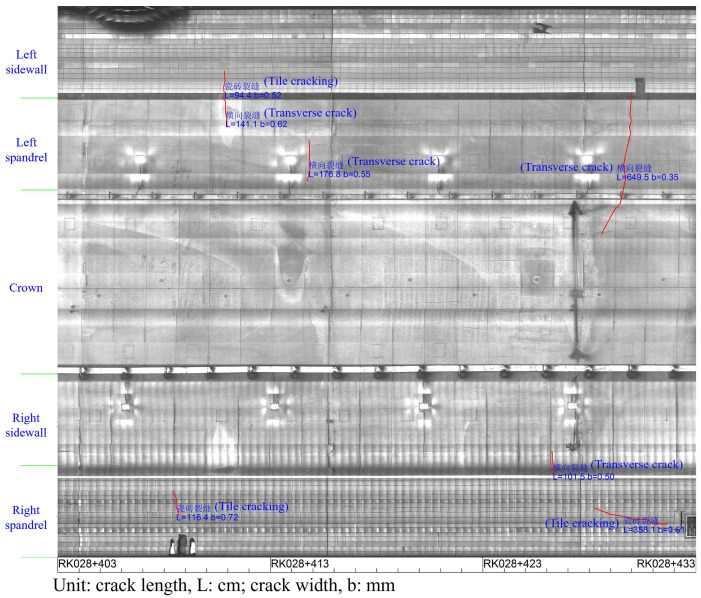
Partial two-dimensional expanded crack maps detected by the tunnel inspection vehicle.

Figure 13 shows representative two-dimensional unfolded crack maps obtained by the inspection equipment. The proposed method can automatically identify and label cracks and calculate their length and width. As shown in Table 4, a total of 411 actual cracks were confirmed during field verification. Among them, the numbers of true positives (TP), false positives (FP), and false negatives (FN) were 408, 49, and 3, respectively. The corresponding Precision, Recall, and F1-score were 89.3%, 99.3%, and 94.0%, respectively. The miss rate was defined as:

(31) $$MissRate=\frac{FN}{TP+FN}.$$

Accordingly, the miss rate was calculated as 0.7%. The numbers of false detections on the fast-lane sidewall, crown region, and slow-lane sidewall were 8, 33, and 8, respectively, indicating that false detections were mainly concentrated in the crown region. This phenomenon is primarily related to the attenuation of infrared illumination intensity with increasing irradiation distance. Because the crown region is generally the farthest from the inspection vehicle, the illumination intensity and uniformity in this area are relatively lower. Consequently, the contrast between short cracks and the lining background may be weakened, and the influence of surface texture and imaging noise on the recognition results may become more pronounced. In addition, interference features such as construction joints and uneven seams may exhibit visual characteristics similar to those of cracks, which can also lead to false detections. Therefore, improving the illumination intensity and uniformity in the crown region, together with increasing the proportion of low-illumination images and easily confused samples in the training dataset, may further enhance the robustness of the proposed recognition method under complex background conditions. Ten cracks with maximum widths ranging from 0.1 to 1.0 mm were randomly selected to compare manual measurements with the algorithm-based results. The comparison is shown in Table 5. Overall, the maximum crack widths calculated by the proposed method agreed well with the manual measurements. The maximum absolute error was 0.12 mm, and the mean relative error was 14.12%. These results indicate that the proposed method has good crack recognition and quantification capability under complex field conditions and has potential for engineering applications.

#### 4.2.3. Water Leakage and Spalling Recognition Test

**Figure 14:**
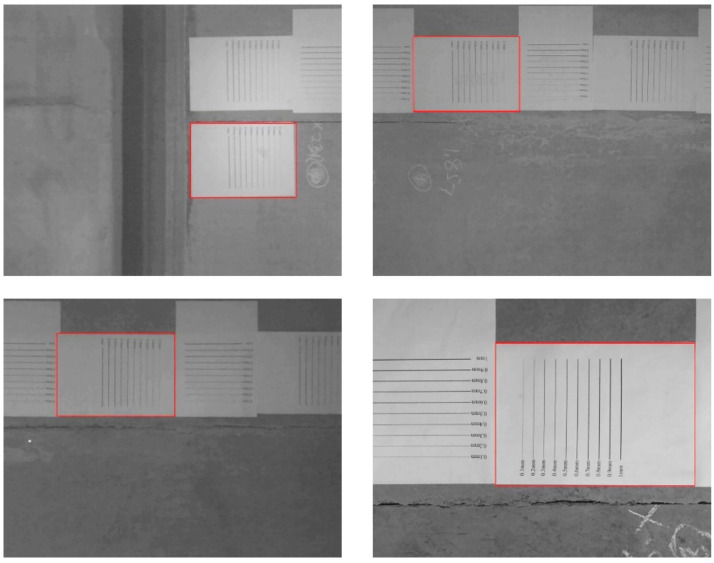
Test results for simulated water leakage and spalling defects.

**Figure 15:**
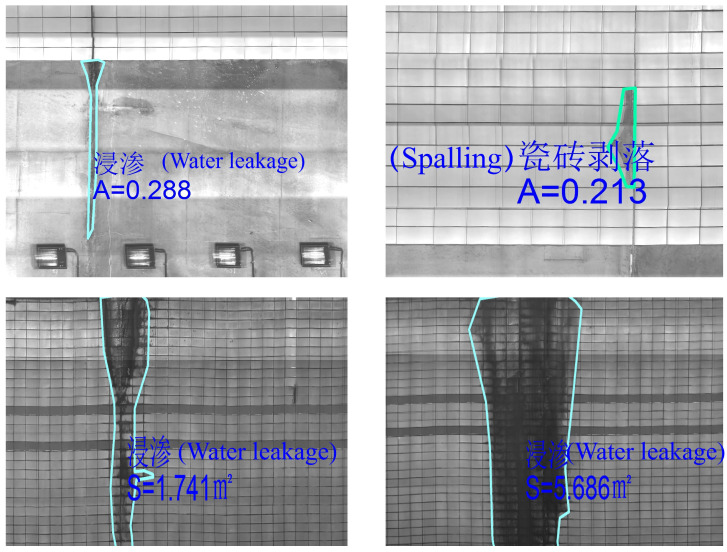
Field recognition results for water leakage and spalling.

As shown in Figure 14, A4 paper sheets with known dimensions were placed at different orientations to quantitatively verify the accuracy of region extraction and area calculation. Image acquisition was performed using the same camera arrangement and imaging procedure as those used in actual tunnel inspection. The A4 paper regions in the acquired images were then identified, and their areas were calculated. As listed in Table 6, the relative area errors of all test regions were less than 0.2%, and the maximum absolute error was 1.2 cm^2^, indicating that the proposed method can achieve high accuracy in controlled region extraction and area calculation. To further evaluate the applicability of the method to real defects with complex textures and irregular boundaries, Figure 15 presents the field recognition results for three water leakage regions and one spalling region. The results show that the proposed method can extract defect regions with different morphologies relatively completely, and the identified boundaries generally agree with the visible defect contours in the field images. This indicates that the method has good adaptability to real water leakage and spalling defects under field conditions.

#### 4.2.4. Tunnel Profile Deformation Test

**Figure 16:**
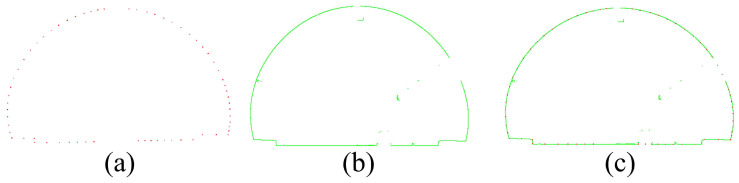
Comparison between total station measurements and rapid inspection equipment results: (**a**) total station measurements; (**b**) inspection equipment results; (**c**) comparison of the two results.

Following the validation approach adopted in previous studies [31,32,33], the effectiveness of the proposed method was evaluated by comparing the profile deformation calculated from the inspection equipment with manual measurements obtained at a representative tunnel cross-section. A Leica TS09 Plus total station was used for the reference survey, with an angular accuracy of 1″ and a distance measurement accuracy of 1.5 mm ± 2 ppm. A full-section survey was performed on the selected tunnel cross-section, and 65 points were measured in total, as shown in Figure 16. The tunnel profiles obtained from the total station and the inspection equipment were then superimposed for comparison, as shown in Figure 16c. Taking the total station measurements as the reference, the deviation was calculated by searching for the nearest point on the profile obtained by the inspection equipment. The maximum nearest-neighbor deviation between the two profiles was 0.28 mm, indicating that the profile obtained by the rapid inspection equipment was generally consistent with the total station measurements and had sufficient reliability for tunnel profile deformation assessment.

## 5. Conclusions

To meet the demand for high-speed mobile inspection of visible defects in highway tunnels, this study established a multisensor inspection and analysis framework integrating high-resolution images, 3D point clouds, and positioning information. The results demonstrate that, through the coordinated design of hardware equipment and intelligent analysis methods, full-section data acquisition, fine-crack recognition, and quantitative assessment of multiple defect types can be achieved at an operating speed of 80 km/h. The main conclusions are as follows.

(1) A mobile inspection system capable of synchronously acquiring full-section tunnel images, 3D point clouds, and mileage information at speeds of up to 80 km/h was developed. The hardware system consists of a vehicle-mounted platform, image acquisition system, laser scanning system, infrared illumination system, mileage positioning module, and data storage module. Compared with existing systems of similar types, the proposed system shows comprehensive advantages in operating speed, fine-crack detection, and integrated inspection of multiple defect types.
(2) A structural-feature-constrained spatial localization method for tunnel defects was established. Stable structural features were used to correct the accumulated error of the mileage encoder, enabling centimetre-level longitudinal localization of defects. By further combining 3D laser cross-sections with the camera mapping relationship, millimetre-level circumferential localization can be achieved. The localization accuracy is affected by the distribution of structural features, recognition quality, and sensor calibration errors.
(3) A recognition and quantification method for multiple visible defects was constructed. Crack detection was achieved by integrating two-stage CNN screening, cascaded segmentation, trajectory tracking, and subpixel edge extraction, while water leakage and spalling were identified using visible–infrared image fusion and adaptive boundary extraction. In the field test, the Precision, Recall, and F1-score for crack recognition were 89.3%, 99.3%, and 94.0%, respectively. Comparisons between field measurements and algorithm-based results for selected cracks indicated that the proposed method can effectively identify cracks with widths as small as 0.1 mm. Controlled experiments and real defect cases further verified the ability of the method to extract irregular defect boundaries and quantify defect areas. Low illumination in the crown region and interference from joints may lead to false or missed detections. Therefore, enhancing crown-region illumination and further optimizing the algorithm using low-illumination and easily confused samples are key to improving recognition reliability.
(4) A tunnel cross-sectional deformation assessment method based on 3D laser point clouds was established. Through tunnel axis reconstruction, point-cloud filtering, and cross-section fitting, a unified coordinate reference was constructed, enabling quantitative calculation of convergence deformation and radial deformation. Field comparisons with total station measurements showed good consistency between the two results, although the calculation accuracy is affected by point-cloud quality and processing errors.
(5) Despite the method’s advantages, challenges remain. The model’s generalization capability requires further evaluation using tunnel-level independent datasets, and the system’s robustness under vehicle vibration, illumination variation, material differences, and local occlusion should be further assessed. In addition, the recognition of irregular boundaries in water leakage and spalling defects, as well as cross-sectional deformation calculation, should be further validated using more field cases, multiple sections, different inspection speeds, and repeated measurements. Future work will focus on tunnel-level independent testing, robustness evaluation, crown-region illumination optimization, and additional field validation.

## Figures and Tables

**Table 1:** Comparison of the proposed mobile inspection system and method with existing studies.

System	System Type	Core Sensors and Auxiliary Devices	Maximum Operating Speed	Minimum Detectable Crack Width	Automatic Detection of Other Defects	Equipment Display
This study	Multisensor	CCD cameras, laser scanner, GPS + encoder, infrared illumination device	80 km/h	0.1 mm	Spalling, water leakage, and tunnel deformation can be detected	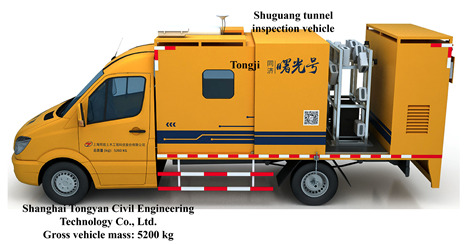
GRP 5000 (Amberg Technologies AG, Regensdorf-Watt, Switzerland) [27]	Single-laser	Laser scanner	0.7 km/h	0.3 mm	Deformation	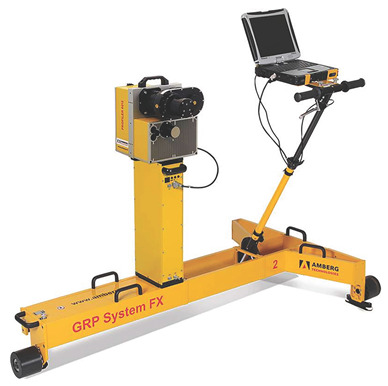
MIMM-R (Pacific Consultants Co., Ltd., Tokyo, Japan) [28]	Multisensor	CCD cameras, laser scanner, GPS + encoder, LED illumination	50 km/h	0.3 mm	Deformation	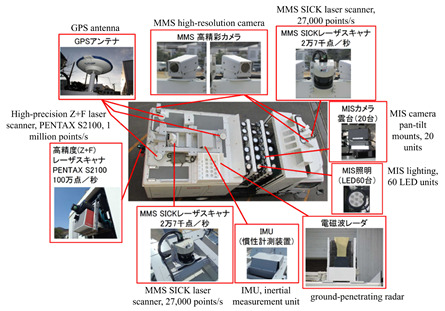
MTI-100 (developed by Tongji University, Shanghai, China) [29]	Single-vision	CCD camera	8.4 km/h	0.3 mm	No	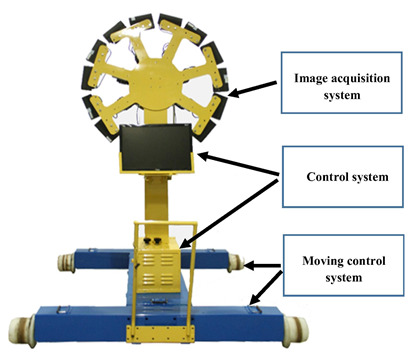
Spacetec TS4 (SPACETEC Datengewinnung GmbH, Freiburg, Germany)	Multisensor	CCD cameras, laser scanner, LED illumination	5 km/h	0.3 mm	No	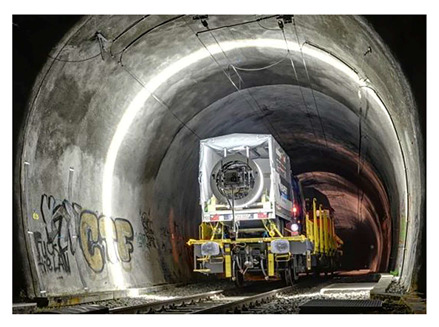
ROBO-SPECT (developed by the ROBO-SPECT Consortium, coordinated by the Institute of Communication and Computer Systems, Athens, Greece) [30]	Multisensor	CCD cameras, laser illumination system, ultrasonic sensor	—	0.5 mm	Spalling	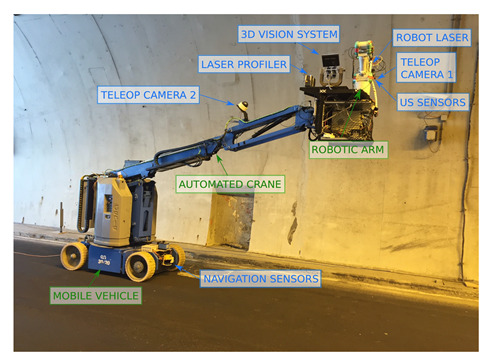
CKY-200 (developed by Wuhan University, Wuhan, China, and Beijing Urban Construction Exploration & Surveying Design Research Institute Co., Ltd., Beijing, China) [9]	Multisensor	CCD cameras, laser scanner, GNSS, illumination system	15 km/h	0.5 mm	No	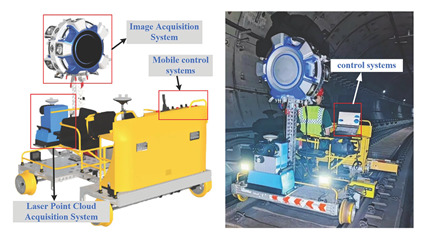
Lightweight tunnel detection equipment (developed by Shandong University and Shandong Research Institute of Industrial Technology, Jinan, China) [10]	Multisensor	CCD cameras, laser scanner, supplementary illumination system	80 km/h	0.5 mm	No	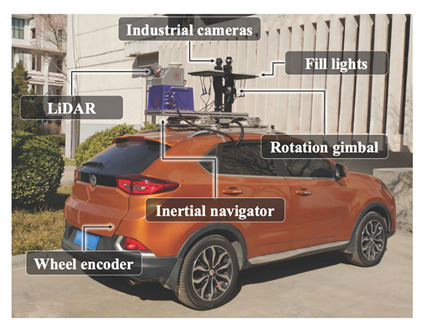

**Table 2:** Network architecture and training parameters of the two-stage CNN.

Parameter	First-Stage CNN	Second-Stage CNN
Purpose	Preliminary crack screening	Suspected crack discrimination
Input image size	512 × 512 pixels	256 × 256 pixels
Number of convolutional layers	10	4
Number of max-pooling layers	4	2
Pooling window size	2 × 2, 4 × 4, 6 × 6, 6 × 6	7 × 7, 4 × 4
Pooling stride	2	2
Activation function	ReLU	ReLU
Output function	Softmax	Softmax
Batch normalization	Yes	Yes
Dropout	Yes	Yes
Optimizer	SGD	SGD
Initial learning rate	0.001	0.001
Momentum	0.9	0.9
Batch size	64	64
Maximum training epochs	2000	2000
Loss function	Categorical cross-entropy loss	Categorical cross-entropy loss
Kernel size	3 × 3, 3 × 3, 3 × 3, 3 × 3, 3 × 3, 3 × 3, 3 × 3, 3 × 3, 3 × 3, 1 × 1	5 × 5, 3 × 3, 3 × 3, 1 × 1
Number of output channels	24, 24, 48, 48, 96, 96, 192, 192, 192, 2	24, 48, 96, 2
Global average pooling	Yes	Yes

**Table 3:** Main settings of the three-stage cascaded crack-path segmentation network.

Category	Parameter Setting
Backbone and feature pyramid	Five-level convolutional backbone; feature map sizes of 128 × 128, 64 × 64, 32 × 32, 16 × 16, and 8 × 8; corresponding channel numbers of 32, 64, 128, 256, and 256; FPN channel number of 128
Anchors and segmentation head	Anchor scales of 8, 16, 32, 64, and 128 pixels, with aspect ratios of 1:3, 1:1, and 3:1; progressive upsampling using 2× transposed convolutions; local probability map size of 32 × 32 pixels
Threshold settings	IoU thresholds of 0.70 and 0.30 for positive and negative anchors, respectively; candidate-box confidence threshold of 0.50; overlap coverage threshold of 0.70; pixel classification threshold of 0.50. These thresholds were determined based on commonly used settings and validation-set performance.
Optimization and training	SGD optimizer; initial learning rate of 0.001; momentum of 0.9; weight decay of 1 × 10^−4^; batch size of 64; maximum of 2000 training epochs; early stopping based on validation-set Dice, with the model achieving the highest validation Dice saved
Data augmentation	Random rotation, brightness adjustment, and Mosaic augmentation
Evaluation metrics	Recall, IoU, and Dice

**Table 4:** Comparison of crack detection results.

Measurement Result	Total Cracks	Fast-Lane Sidewall	Crown Region	Slow-Lane Sidewall	Circumferential Cracks	Longitudinal Cracks	Inclined Cracks
Correct detections	408	86	179	143	186	130	92
False detections	49	8	33	8	19	13	17

**Table 5:** Comparison between manually measured and algorithm-calculated maximum crack widths.

Crack No.	Manually Measured Maximum Width (mm)	Algorithm-Calculated Maximum Width (mm)	Absolute Error (mm)	Relative Error (%)
1	0.25	0.28	0.03	12.00
2	0.10	0.13	0.03	30.00
3	0.51	0.59	0.08	15.69
4	0.22	0.28	0.06	27.27
5	0.69	0.60	0.09	13.04
6	0.50	0.52	0.02	4.00
7	0.51	0.58	0.07	13.73
8	0.89	0.82	0.07	7.87
9	1.00	0.88	0.12	12.00
10	0.71	0.67	0.04	5.63

**Table 6:** Comparison of defect area measurement results.

Group	Actual Area A_s_ (mm^2^)	Calculated Area A_c_ (mm^2^)	Area Difference A_T_ (mm^2^)	Error (%)
1	62,370	62,286	84	−0.135
2	62,370	62,398	28	0.045
3	62,370	62,420	−50	0.080
4	62,370	62,250	120	−0.192
5	62,370	62,450	−80	0.128
6	62,370	62,282	88	−0.141
7	62,370	62,470	−100	0.160

## Data Availability

The data presented in this study are available from the corresponding author upon reasonable request. The raw data are not publicly available because they are subject to project data-management and engineering-site confidentiality restrictions.

## References

[1] Attard L., Debono C.J., Valentino G., Di Castro M. (2018). Tunnel Inspection Using Photogrammetric Techniques and Image Processing: A Review. ISPRS J. Photogramm. Remote Sens..

[2] Erarslan N. (2019). Analysing Mixed Mode (I–II) Fracturing of Concrete Discs Including Chevron and Straight-through Notch Cracks. Int. J. Solids Struct..

[3] Liu C., Zhang D., Zhang S. (2021). Characteristics and Treatment Measures of Lining Damage: A Case Study on a Mountain Tunnel. Eng. Fail. Anal..

[4] Tonon F. (2010). Sequential Excavation, NATM and ADECO: What They Have in Common and How They Differ. Tunn. Undergr. Space Technol..

[5] Zhang X., Li M., Tang L., Memon S.A., Ma G., Xing F., Sun H. (2017). Corrosion Induced Stress Field and Cracking Time of Reinforced Concrete with Initial Defects: Analytical Modeling and Experimental Investigation. Corros. Sci..

[6] Liu X., Hong J., Liu Z., Cao B.T. (2026). Mechanical Behavior and Failure Mechanisms of Shield Tunnel Linings Reinforced with Lightweight Epoxy Bonded-Bolted Steel Plates. Tunn. Undergr. Space Technol..

[7] Liu X., Hong J., Liu Z. (2025). Investigations on Mechanical Behavior of Longitudinal Joints in Segmental Tunnel Linings Reinforced with Epoxy Bonded-Bolted Steel Plates. Tunn. Undergr. Space Technol..

[8] Dang L.M., Wang H., Li Y., Park Y., Oh C., Nguyen T.N., Moon H. (2022). Automatic Tunnel Lining Crack Evaluation and Measurement Using Deep Learning. Tunn. Undergr. Space Technol..

[9] Fan T., Mao Q., Tang C., Wang L., Li J., Bao Y., Gao T., Pei L., Zhang D., Wang Y. (2025). Rapid Mobile Inspection Equipment for Metro Tunnels Based on Multi-Sensor Integration. Sci. Rep..

[10] Liu J., Lv C., Lu G., Zhao Z., Han B., Guo F., Xie Q. (2024). Lightweight Defect Detection Equipment for Road Tunnels. IEEE Sens. J..

[11] Xu H., Wang M., Liu C., Li F., Xie C. (2024). Automatic Detection of Tunnel Lining Crack Based on Mobile Image Acquisition System and Deep Learning Ensemble Model. Tunn. Undergr. Space Technol..

[12] Otsu N. (1979). A Threshold Selection Method from Gray-Level Histograms. IEEE Trans. Syst. Man. Cybern..

[13] Wang G., Tse P.W., Yuan M. (2018). Automatic Internal Crack Detection from a Sequence of Infrared Images with a Triple-Threshold Canny Edge Detector. Meas. Sci. Technol..

[14] Li C., Xu P., Niu L., Chen Y., Sheng L., Liu M. (2019). Tunnel Crack Detection Using Coarse-to-Fine Region Localization and Edge Detection. WIREs Data Min. Knowl. Discov..

[15] Ai D., Jiang G., Lam S.-K., He P., Li C. (2023). Computer Vision Framework for Crack Detection of Civil Infrastructure—A Review. Eng. Appl. Artif. Intell..

[16] Han W., Jiang Y., Wang G., Liu C., Koga D., Luan H. (2023). Review of Health Inspection and Reinforcement Design for Typical Tunnel Quality Defects of Voids and Insufficient Lining Thickness. Tunn. Undergr. Space Technol..

[17] Gao X., Ding Z., Shi S., Zhou J., Huang P., Zheng H., Wang C. (2023). Auto-Inspection System Using Optimized Fuzzy Sliding Mode Control Strategy for Tunnel Inspection. Electronics.

[18] Sasama H., Ukai M., Ohta M., Miyamoto T. (1998). Inspection System for Railway Facilities Using a Continuously Scanned Image. Electr. Eng. Jpn..

[19] Liu Y.-F., Nie X., Fan J.-S., Liu X.-G. (2020). Image-Based Crack Assessment of Bridge Piers Using Unmanned Aerial Vehicles and Three-Dimensional Scene Reconstruction. Comput.-Aided Civ. Infrastruct. Eng..

[20] Zheng Y., Gao Y., Lu S., Mosalam K.M. (2022). Multistage Semisupervised Active Learning Framework for Crack Identification, Segmentation, and Measurement of Bridges. Comput.-Aided Civ. Infrastruct. Eng..

[21] Zhou Z., Zhang J., Gong C. (2022). Automatic Detection Method of Tunnel Lining Multi-Defects via an Enhanced You Only Look Once Network. Comput.-Aided Civ. Infrastruct. Eng..

[22] Sjölander A., Belloni V., Ansell A., Nordström E. (2023). Towards Automated Inspections of Tunnels: A Review of Optical Inspections and Autonomous Assessment of Concrete Tunnel Linings. Sensors.

[23] Hacıefendioğlu K., Demir S. (2026). Deep Learning–Based Automated Crack Detection for Post-Earthquake Damage Assessment in Reinforced Concrete Structures. Adv. Civ. Eng..

[24] Gu W., Liu X., Li Z. (2024). Sustainable Infrastructure Maintenance: Crack Depth Detection in Tunnel Linings via Natural Temperature Variations and Infrared Imaging. Sustainability.

[25] Zheng A., Qi S., Cheng Y., Wu D., Zhu J. (2024). Efficient Detection of Apparent Defects in Subway Tunnel Linings Based on Deep Learning Methods. Appl. Sci..

[26] Sun H., Xu Z., Yao L., Zhong R., Du L., Wu H. (2020). Tunnel Monitoring and Measuring System Using Mobile Laser Scanning: Design and Deployment. Remote Sens..

[27] Engstrand A. (2011). Railway Surveying—A Case Study of the GRP 5000. Master’s Thesis.

[28] Yasuda T., Yamamoto H., Shigeta Y. (2016). Tunnel Inspection System by Using High-Speed Mobile 3D Survey Vehicle: MIMM-R. J. Robot. Soc. Jpn..

[29] Huang H., Sun Y., Xue Y., Wang F. (2017). Inspection Equipment Study for Subway Tunnel Defects by Grey-Scale Image Processing. Adv. Eng. Inf..

[30] Menendez E., Victores J.G., Montero R., Martínez S., Balaguer C. (2018). Tunnel Structural Inspection and Assessment Using an Autonomous Robotic System. Autom. Constr..

[31] Xie X., Lu X. (2017). Development of a 3D Modeling Algorithm for Tunnel Deformation Monitoring Based on Terrestrial Laser Scanning. Undergr. Space.

[32] Gikas V. (2012). Three-Dimensional Laser Scanning for Geometry Documentation and Construction Management of Highway Tunnels during Excavation. Sensors.

[33] Camara M., Wang L., You Z. (2024). Tunnel Cross-Section Deformation Monitoring Based on Mobile Laser Scanning Point Cloud. Sensors.

